# A147 FACTORS ASSOCIATED WITH PATIENT COMFORT DURING ENDOSCOPIC RESECTION OF LARGE NON-PEDUNCULATED COLORECTAL POLYPS

**DOI:** 10.1093/jcag/gwad061.147

**Published:** 2024-02-14

**Authors:** A Zarrin, S X Jiang, A Walia, C Galorport, W Xiong, J Telford, R Enns, E Lam, N Shahidi

**Affiliations:** The University of British Columbia Faculty of Medicine, Vancouver, BC, Canada; The University of British Columbia Faculty of Medicine, Vancouver, BC, Canada; St. Paul's Hospital, Division of Gastroenterology, Vancouver, BC, Canada; St. Paul's Hospital, Division of Gastroenterology, Vancouver, BC, Canada; University of British Columbia, Department of Pathology and Laboratory Medicine, Vancouver, BC, Canada; St. Paul's Hospital, Division of Gastroenterology, Vancouver, BC, Canada; St. Paul's Hospital, Division of Gastroenterology, Vancouver, BC, Canada; St. Paul's Hospital, Division of Gastroenterology, Vancouver, BC, Canada; St. Paul's Hospital, Division of Gastroenterology, Vancouver, BC, Canada

## Abstract

**Background:**

Patient comfort is an important quality indicator in endoscopy, as it relates to patient satisfaction and adherence with endoscopic surveillance recommendations. Endoscopic resection techniques are now the primary treatment strategy for LNPCPs. However, factors associated with patient comfort are unknown.

**Aims:**

To assess patient comfort during endoscopic resection of LNPCPs and factors associated with discomfort.

**Methods:**

Consecutive patients ampersand:003E 18 years of age who underwent endoscopic resection for a LNPCP were enrolled in a prospective single center observation cohort study (clinicaltrials.gov ID: NCT05402696). The St. Paul’s Endoscopic Comfort Scale (SPECS) was used to assess patient comfort (total score; vocalization, body language, anxiety sub-scores). Patient discomfort was defined as a SPECS total or sub-score above 0.

**Results:**

Between 06/2022 - 07/2023, 258 patients underwent 276 procedures to remove 318 LNPCPs. Median age was 67.0 years and 126 (48.9%) were female. Most patients were ASA II (168, 57.3%) and 268 (97.1%) received conscious sedation with midazolam and/or fentanyl. Median SPECS score was 0 (IQR 1) with a range of 0 (145, 56.6%) to 5 (2, 0.8%). Challenging access and proximal LNPCP location were associated with overall discomfort and vocalization respectively (both p ampersand:003C 0.03). Highest rate of discomfort corresponded to lesions with difficult reach and easy positioning (70.6% of patients with these lesions had SPECSampersand:003E0). Lesion size, resection technique or resection duration were not associated with discomfort.

**Conclusions:**

Minimally invasive endoscopic resection techniques can be performed without patient discomfort in most patients with LNPCPs. Challenging access and proximal location should be taken into consideration when administering sedation.

**Funding Agencies:**

None

Gastrointestinal Oncology

